# PAGE: Parametric Analysis of Gene Set Enrichment

**DOI:** 10.1186/1471-2105-6-144

**Published:** 2005-06-08

**Authors:** Seon-Young Kim, David J Volsky

**Affiliations:** 1 Molecular Virology Division, St. Luke's-Roosevelt Hospital Center and Columbia University, New York, NY 10019, USA; 2 Human Genomics Laboratory, Genome Research Center, Korea Research, Institute of Biosciences and Biotechnology 52 Eoeun-dong Yuseong-gu, Daejon, 305-333, Korea; 3 Molecular Virology Division, St. Luke's-Roosevelt Hospital Center 432 West 58th, Street Antenucci Building, Room 709 New York, NY 10019, USA

## Abstract

**Background:**

Gene set enrichment analysis (GSEA) is a microarray data analysis method that uses predefined gene sets and ranks of genes to identify significant biological changes in microarray data sets. GSEA is especially useful when gene expression changes in a given microarray data set is minimal or moderate.

**Results:**

We developed a modified gene set enrichment analysis method based on a parametric statistical analysis model. Compared with GSEA, the parametric analysis of gene set enrichment (PAGE) detected a larger number of significantly altered gene sets and their p-values were lower than the corresponding p-values calculated by GSEA. Because PAGE uses normal distribution for statistical inference, it requires less computation than GSEA, which needs repeated computation of the permutated data set. PAGE was able to detect significantly changed gene sets from microarray data irrespective of different Affymetrix probe level analysis methods or different microarray platforms. Comparison of two aged muscle microarray data sets at gene set level using PAGE revealed common biological themes better than comparison at individual gene level.

**Conclusion:**

PAGE was statistically more sensitive and required much less computational effort than GSEA, it could identify significantly changed biological themes from microarray data irrespective of analysis methods or microarray platforms, and it was useful in comparison of multiple microarray data sets. We offer PAGE as a useful microarray analysis method.

## Background

High-throughput technologies such as DNA microarrays and proteomics are revolutionizing biology and medicine. Global gene expression profiling using microarrays monitors changes in expression of thousands of genes simultaneously. At the data acquisition level, gene expression profiles from a given system should be reproducible and yield statistically significant changes in gene expression [1]. The large amounts of data acquired must then be reduced or "translated" to a smaller set of genes representing meaningful biological differences between control and test systems and validated in an experimental or clinical setting [2]. Since inception of the microarray technology, significant technological and analytical improvements have been introduced to meet these challenges, from experimental design [1], probe-level analysis of oligonucleotide chips [3,4], data normalization [5], statistical analysis [6], clustering techniques [7-9], to various data mining tools [10-12]. A large number of studies used microarrays successfully to discern changes in gene expression patterns either in well defined cellular populations responding to a specific stimulus in vitro [13,14] or in complex clinical settings such as cancer and neurological diseases [15-17].

While individual microarray studies can be highly informative, it is generally difficult to compare independently obtained data sets addressing the same biological problem [18], regardless of whether the same or different microarray platform was used [19-23]. The poor congruence of cross-study comparisons was attributed to incorrectly annotated probes, non-sequence overlapping but Unigene-matching probes, variation in experimental conditions, and actual biological variations among different clinical or experimental materials used [19-23]. Several new bioinformatics programs have attempted to circumvent the variability between different published data sets at the data acquisition level by comparing gene expression results for coordinate changes in biological themes [12], for similarity of significance values for each gene obtained through meta-analysis methods [15], and for reproducible gene expression patterns revealed by integrative correlation statistics [24]. Each of the methods revealed some congruency among the data sets analyzed [12,15,24] indicating that results from various transcriptional profiling studies can eventually be integrated for better general definition of normal and disease-related processes.

Another challenge of microarray data analysis is that the majority of genes in any genome-wide transcriptional profile data set are excluded from consideration because they show only subtle changes in expression. This problem was recently addressed in a gene expression profiling study of human diabetic muscles, in which no single gene (out of over 20,000) showed significant difference in expression between control and patients groups [25]. Assuming that gene expression changes can be detected at the level of co-regulated gene sets rather than individual genes, the authors devised a new analytical tool, GSEA, that tested predefined gene sets for association with disease phenotypes [25]. GSEA successfully detected oxidative phosphorylation as a biological theme that coordinately changed in diabetic muscles [25]. Although subsequent analysis suggested that the statistical tools used in GSEA may be biased toward assigning higher enrichment scores to gene sets of large size [26], the program significantly expands the potential for discovery of important process or disease related genes in a given microarray data set.

In the present work we describe a modified gene set enrichment analysis strategy that improves analysis of minimally changed gene expression profiles. PAGE employs fold change between experimental groups or other parametric data to calculate Z scores of predefined gene sets and use normal distribution to infer statistical significance of gene sets. We show here that PAGE has several advantages over GSEA and is useful in comparison of multiple microarray data sets.

## Results

### Statistical model and selection of minimal gene set size

According to the Central Limit Theorem in statistics, the distribution of the average of randomly sampled n observations tends to follow normal distribution as the sampling size n becomes larger, even when the parent distribution from which the average is calculated is not normal. The distribution of the average of randomly sampled observations has the same mean as the parent distribution and its variance is equal to the variance of the parent divided by the sampling size [27]. In other words, when the mean and variance of the parent distribution (whether it is normally distributed or not) are μ and σ^2 ^the average of n observations from the parent distribution will follow a normal distribution of mean μ and variance σ^2^/n when the sampling size n is large enough. In PAGE, the parent distribution is a distribution of any numerical values (also termed parameters here) that describe differential expression of genes among samples in a microarray data set. Usually, the values are a fold change for an individual gene between two experimental groups or they can be a correlation coefficient between clinical indices and individual gene expression values in a microarray data set. In most cases, the distribution of a parameter, i.e., a fold change values for all genes in a gene set between two experimental groups, is not normally distributed. However, as the Central Limit Theorem states, when we sample n observations from the parent distribution of a parameter, the average of the sampled observations tends to follow the normal distribution as our sampling size n becomes larger. Here, we define sampled observations as expression values for randomly chosen individual genes within pre-defined gene sets, which may be any randomly chosen groups of genes, groups of genes representing close family members with similar functions, genes in the same biological pathway, and so on. If we define a gene set of sufficiently large size, we can use the normal distribution to test the statistical significance of that gene set.

**Figure 1:**
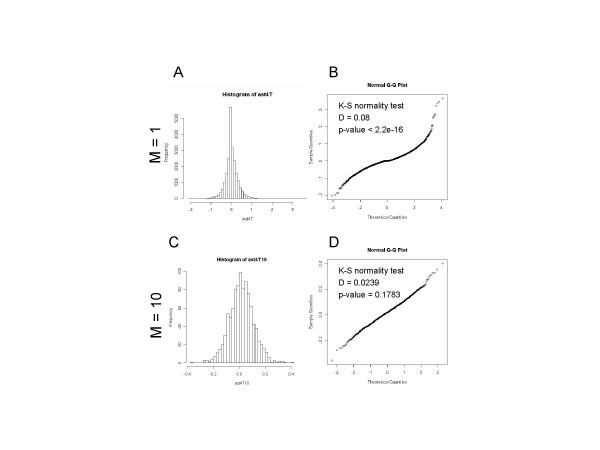
**Distribution pattern of fold change values in a microarray data and determination of minimal gene set size in PAGE. **A and B. A Histogram (A) and a quantile-quantile (Q-Q) plot against standard normal distribution (B) of fold change values from microarray data set. The diabetic muscle microarray data set [25] was analyzed as described in Methods section. The fold change values between normal and patient groups were calculated and used to draw histogram (A) and Q-Q plot (B). C and D. A Histrogram (C) and a Q-Q plot (D) of an average of 10 randomly sampled values from fold change values of diabetic muscle microarray data. Kolmogorov-Smirnov normality test was performed with a null hypothesis that distribution is normal. For the distribution of fold change values (A and B), the null hypothesis was rejected (D = 0.08, p-value < 2.2e-16). For the distribution of an average of 10 randomly sampled values from fold change values (C and D), the null hypothesis was not rejected (D = 0.0239, p-value = 0.1783).

To determine the minimal gene set size m, we first examined the distribution pattern of several microarray data sets. We used fold change between two experimental groups as a parameter and observed the distribution of fold change values in a microarray data set. As an example, we show the distribution of fold change values from microarray data set that compared gene expression of diabetic muscles with that of normal control muscles [25] (Fig. 1). The histogram of fold change values (Fig. 1A) and quantile-quantile plot of fold change values against standard normal distribution (Fig. 1B) suggested that fold change values were not normally distributed. The null hypothesis that the distribution of fold change values was normal was rejected by Kolmogorov-Smirnov normality test (D = 0.08, p-value < 2.2e-16).

Subsequently, we sampled m genes from the parent population of fold change values, calculated the average of the sampled m observations, and observed the distribution pattern of the average values. We began from sampling size two, incremented by one until the sampling size m became 50. As expected, as the sampling size m increased, we found that the distribution of the average of sampled observations became closer to normal. As an example, we show the distribution of the average with sampling size of 10 (Fig. 1C and 1D). Both histogram (Fig. 1C) and quantile-quantile plot against standard normal distribution (Fig. 1D) suggested that the distribution of the average of 10 observations was close to normal and the null hypothesis that this distribution was normal was not rejected by Kolmogorovo-Smirnov normality test (D = 0.0239, p-value = 0.1783). Based on these observations, we set the minimal gene set size as 10. We performed the same analysis with several microarray data sets using diverse data processing procedures and found similar results (data not shown).

### Comparison of PAGE with GSEA

GSEA calculates an enrichment score (ES) for a given gene set using rank of genes and infers statistical significance of each ES against ES background distribution calculated by permutation of the original data set. In contrast, PAGE calculates a Z score for a given gene set from a parameter such as fold change value between two experimental groups and infers statistical significance of the Z score against standard normal distribution.

To compare the statistical sensitivity of PAGE with that of GSEA, we analyzed human diabetic muscle microarray data sets used in the initial description of GSEA [25]. We calculated fold change values between diabetic muscles and control muscles as described above, calculated Z scores and corresponding p-values for each gene set, and compared these parameters with enrichment scores and corresponding p-values available as supplementary information (Table 1). We found that both PAGE and GSEA detected OXPHOS_HG-U133A as the most significantly changed gene set; however, the statistical significance of PAGE detection of this gene set was much greater, p = 1 × 10^-11 ^versus p = 0.003. PAGE and GSEA ranked the next three gene sets, human_mitoDB_6_2002_HG-U133A, mitochondr_HG-U133A, and MAP00190_Oxidative_Phosphorylation, as the second through fourth significant gene sets and again, the statistical power of PAGE analysis (the p-value) was greater than that of GSEA (Table 1). Overall, PAGE detected seven gene sets as statistically significant at p < 0.05 in this data set whereas GSEA detected only one gene set at this significance. For all gene sets, p-values obtained by PAGE analysis PAGE were generally smaller than p-values of corresponding gene sets obtained by GSEA.

**Table 1:** Comparison of PAGE with GSEA

**PAGE**			**GSEA**		
Gene Set	Z score	p-value	Gene Set	ES	p-value
OXPHOS_HG-U133A	-10.5835	<1.0E-11	OXPHOS_HG-U133A	346.8827	0.003
human_mitoDB_6_2002_HG-U133A	-6.7213	1.81E-11	human_mitoDB_6_2002_HG-U133A	215.9424	0.091
mitochondr_HG-U133A	-6.4761	9.46E-11	mitochondr_HG-U133A	207.9381	0.087
MAP00190_Oxidative_phosphorylation	-4.5745	4.78E-05	c20_U133	181.1569	0.062
c20_U133	-3.7461	0.0002	MAP00190_Oxidative_phosphorylation	148.9061	0.084
c25_U133	-2.7617	0.0058	c22_U133	142.9006	0.028
c21_U133	-2.1116	0.0347	c29_U133	131.4732	0.026

We extended the comparison of PAGE and GSEA to additional data sets including gene expression profiles of young and aged muscles from males (GDS 287) and females (GDS 472), or dermal fibroblasts subjected or not subjected to oxidative stress (GDS 963n1). Unlike the analysis shown in Table 1, we applied GSEA here as a multiple comparison testing tool [28] to directly compare the ability of both programs to detect multiple significant gene sets. The results of these analyses are shown in Additional files 1, 2, 3, 4. In data set GDS 472, PAGE detected 15 out of the first 30 gene sets as significantly up-regulated versus 1 out of 30 for GSEA (see Additional file 1). The corresponding numbers in GDS 287 were 26 of 30 for PAGE and 12 of 30 for GSEA (see Additional file 2). In data set GDS 963n1, both PAGE and GSEA detected 14 significant gene set out of 32 (see Additional file 3). It should be noted that the results in Additional files 1, 2, 3 were ranked by the GSEA NE scores. Although in many cases the same gene sets were identified as significant by both programs, PAGE generally detected a larger number of significant gene sets than GSEA across the entire range of GSEA NES. This is illustrated by expressing the results of the analysis shown in Additional file 3 with gene set rankings by the PAGE Z score (see Additional file 4). These results further demonstrate the utility of PAGE for sensitive detection of significantly altered biological pathways in various publicly available microarray data sets.

To further compare statistical robustness of PAGE and GSEA for detection of minimally changed gene sets we performed a simulation study using 10 hypothetical "experimental" and 10 "control" data sets, each containing expression values for 2,000 genes that were randomly chosen from standard normal distribution curve (see Additional file 5). Of the 2000 genes, 20 were designated as a hypothetical gene set of interest. The method and parameters of this simulation are described in the legend to Additional file 5. The simulation indicates that PAGE is more statistically sensitive than GSEA, being able to detect the test gene set as significant when the mean difference between the "experiment" and "control" was as small as 0.25 (see Additional file 5).

### Robustness of PAGE across different microarray probe analysis methods or different microarray platforms

For a given microarray data set, use of different methods of data preprocessing, array normalization, and statistical inference can lead to different end results [3,4,29]. We tested the general applicability of PAGE to different Affymetrix probe analysis programs using the Duchenne muscular dystrophy (DMD) data set GDS 563 which contains 29 CEL files available from Gene Expression Omnibus website. Starting with 11 control and 23 DMD sets, we calculated expression values by MAS5 [30], MBEI [3], and RMA [4] programs, logarithm transformed expression values calculated by MAS5 and MBEI by base two, determined fold changes between two groups, and performed PAGE with pathway gene sets. With a cut-off p-value of < 0.05, PAGE identified eight significantly impaired gene sets with MAS5 and RMA platforms and six suppressed gene sets with MBEI platform, although the next three gene sets with lower significance derived from MBEI analysis were the same as those identified with MAS5 or RMA platforms (Table 2). For significantly induced gene sets, all three methods identified identical five gene sets (Table 2).

**Table 2:** Application of PAGE to different Affymetrix probe level analysis methods

MAS5			MBEI			RMA		
Gene Set	Z score	p-value	Gene Set	Z score	p-value	Gene Set	Z score	p-value

Inflammatory Response Pathway	7.5051	<1.0E-12	Inflammatory Responses	7.5195	5.51E-14	Inflammatory Responses	7.3487	2.00E-13
Eicosanoid Synthesis	6.5925	<1.0E-12	Eicosanoid Synthesis	3.8957	9.79E-05	Eicosanoid Synthesis	4.1557	3.24E-05
Complement Activation Classical	3.0382	0.0024	Complement Activation	3.5487	0.0004	TGF-β Signaling Pathway	3.0438	0.0023
Nucleotide Metabolism	2.0536	0.0400	Nucleotide Metabolism	2.4207	0.0155	Complement Activation Classical	2.9402	0.0033
TGF-β Signaling Pathway	1.9758	0.0482	TGF-β Signaling Pathway	2.2379	0.0250	Nucleotide Metabolism	2.5867	0.0097
								
MAPK Cascade	-2.2529	0.0243	Glutamate Metabolism	-1.7249	0.0846	GPCRs Class A Rhodopsin-like	-1.9907	0.0465
Translation Factors	-2.3124	0.0208	MAPK Cascade	-1.8685	0.0617	MAPK Cascade	-2.1306	0.0331
Krebs-TCA Cycle	-3.2551	0.0011	Proteasome Degradation	-1.9605	0.0499	Krebs-TCA Cycle	-2.6076	0.0091
Glycogen Metabolism	-3.3488	0.0008	Krebs-TCA Cycle	-2.1182	0.0342	Proteasome Degradation	-2.7822	0.0054
Proteasome Degradation	-3.7468	0.0002	Glycogen Metabolism	-2.8330	0.0046	Glycogen Metabolism	-2.9328	0.0034
Fatty Acid Degradation	-3.8286	0.0001	Fatty Acid Degradation	-2.8570	0.0043	Fatty Acid Degradation	-3.4024	0.0007
Nuclear Receptors	-4.2579	2.06E-05	Nuclear Receptors	-3.4686	0.0005	Nuclear Receptors	-4.1600	3.18E-05
Electron Transport Chain	-6.3789	1.78E-10	Electron Transport Chain	-4.2009	2.66E-05	Electron Transport Chain	-5.1177	3.09E-07

We then tested whether PAGE could detect common biological themes from microarray data sets produced using different microarray platforms. We analyzed breast cancer cell line experiment GSE 1299 in which three different arrays (U133A, U95A, and Agilent Human cDNA) were employed with the same RNA to analyze platform dependency of microarray data [20]. We programmed PAGE to identify pathway gene sets in each of the data sets obtained by three microarray platforms used in the experiment. Among significantly down-modulated pathways, two pathways (gap_junction proteins-connexins and cholesterol_biosynthesis) were identified by PAGE as common to all three microarray platforms (Table 3) and for up-regulated gene sets, Krebs_TCA was detected with all three microarray platforms (Table 3).

**Table 3:** Comparison of PAGE results from data sets produced using different microarray platforms

U95A			U133A			Agilent		
Gene Set	Z score	p-value	Gene Set	Z score	p-value	Gene Set	Z score	p-value

Complement Activation Classical	3.0477	0.0023	Krebs TCA Cycle	5.0505	4.41E-07	tRNA Synthetases	3.1714	0.0015
Krebs-TCA Cycle	2.6771	0.0074	Cell Cycle	3.4088	0.0007	Krebs TCA Cycle	3.1291	0.0018
Nuclear Receptors	2.6562	0.0079	Translation Factors	2.8213	0.0048	Proteasome Degradation	1.9894	0.0467
Calcium Channels	1.6956	0.0900	Nuclear Receptors	2.3404	0.0193	Glycolysis and Gluconeogenesis	1.5679	0.1169
Apoptosis	1.4085	0.1590	Complement Activation Classical	2.2861	0.0223	Steroid Biosynthesis	1.5109	0.1308
								
TGF Beta Signaling Pathway	-1.8580	0.0632	Matrix Metalloproteinases	-2.2837	0.0224	Ribosomal Proteins	-2.1275	0.0334
Inflammatory Response Pathway	-2.2590	0.0239	TGF Beta Signaling Pathway	-3.3762	0.0007	Glycogen Metabolism	-2.4904	0.0128
Glycogen Metabolism	-2.6981	0.0070	Inflammatory Response Pathway	-3.5668	0.0004	TGF Beta Signaling Pathway	-2.6910	0.0071
Cholesterol Biosynthesis	-4.6988	2.62E-06	Cholesterol Biosynthesis	-5.8165	6.01E-09	Cholesterol Biosynthesis	-4.1642	3.12E-05
Gap Junction Proteins-Connexins	-5.7792	7.51E-09	Gap Junction Proteins-Connexins	-6.2689	3.64E-10	Gap Junction Proteins-Connexins	-6.6162	3.69E-11

### Application of PAGE to comparing different microarray data sets

Comparison of different microarray data sets dealing with similar biological questions often poses a problem of poor congruency among data sets when compared at gene level. We tested whether comparing at gene set level was better than at individual gene level to reveal congruency among different microarray data sets.

**Figure 2:**
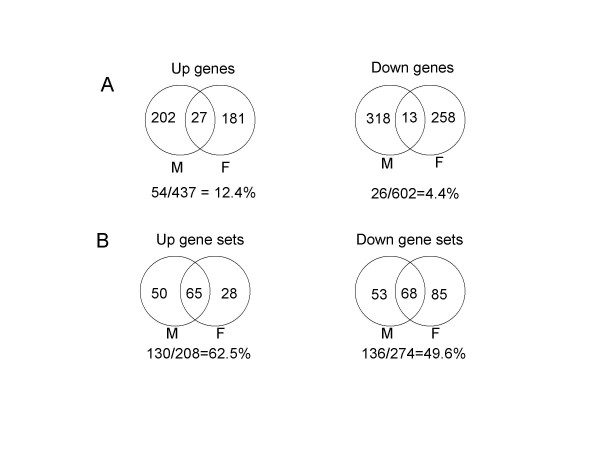
**Comparison of different microarray data sets at gene set level shows better congruence than comparison at gene level. **A. Comparison of two different microarray data sets at gene level. Two microarray data sets, GDS 287 (Muscle function and aging-Male) and GDS 472 (Muscle function and aging-Female) were analyzed, significantly changed genes (|fold change| > 1.5 and t-test p < 0.05) from each data set were selected, and the percentage of common gene lists for both data sets was calculated. B. Comparison at gene set level. We first performed PAGE on the two microarray data sets, selected significant gene sets (p < 0.05), and calculated percentage of common gene sets for both data sets.

We compared two microarray data sets, GDS 287 and GDS 472, produced by the same authors using the same microarray platform Affymetrix U133A (Fig. 2 and Table 4). The data set GDS 287 records differential gene expression of muscles of young and old aged males, and GDS 472 records differential gene expression of muscles of young and old aged females. We analyzed both data sets in the same manner, selected significantly changed genes (|fold change| > 1.5 and t-test p-value < 0.05), and found the percentage of genes common to both data sets. Only 12.4% of significantly up-regulated genes occurred in both data sets and 4.4% of significantly down-regulated genes occurred in both data sets (Fig. 2A). In contrast, when we compared both data sets at gene set level, 62.5% of significantly up-regulated gene sets occurred in both data sets and 49.6% of significantly down-regulated gene sets occurred in both data sets (Fig. 2B). Actually, gene set level comparison correctly pointed out that energy metabolism such as electron transport, tricarboxylic acid cycle, and glycolysis was impaired and genes involved in mRNA processing and cell cycle regulation were up-regulated in both old aged male (GDS 287) and old aged female (GDS 472) data sets (Table 4).

**Table 4:** Comparison of two microarray data sets at gene set level

	GDS 287		GDS 472	
Gene Set	Z score	p-value	Z score	p-value
mRNA processing	4.0322	0.0001	3.2288	0.0012
cell cycle	3.0715	0.0021	3.1127	0.0019
mRNA catabolism	2.2233	0.0262	2.9762	0.0029
mRNA splicing	5.9613	3.00E-09	2.8041	0.0050
nuclear mRNA splicing_via spliceosome	6.3123	2.75E-10	2.6642	0.0077
regulation of cyclin dependent protein kinase activity	2.1343	0.0328	2.5703	0.0102
G1 phase of mitotic cell cycle	2.5482	0.0108	2.5361	0.0112
negative regulation of cell proliferation	3.0116	0.0026	2.0812	0.0374
cholesterol metabolism	2.2758	0.0229	2.0312	0.0422
				
blood coagulation	-2.6705	0.0076	-2.0119	0.0442
protein folding	-2.1739	0.0297	-2.1045	0.0353
regulation of blood pressure	-3.0984	0.0019	-2.3543	0.0186
carboxylic acid transport	-2.9366	0.0033	-2.8088	0.0050
signal transduction	-2.5917	0.0095	-3.0115	0.0026
glycolysis	-4.0631	4.84E-05	-5.3157	1.06E-07
tricarboxylic acid cycle	-2.2914	0.0219	-6.3019	2.94E-10
electron transport	-3.9150	0.0001	-6.9442	3.81E-12

## Discussion

Our initial objectives in developing PAGE were to increase the statistical power of the existing gene set enrichment program for analysis of subtle changes in microarray data and to simplify the laborious computational process involved. We designed PAGE as a parametric statistical test that uses normal distribution to infer the statistical significance of Z scores calculated from actual numerical parameters such as fold change between two experimental groups. Distribution-free, non-parametric methods such as the ones used in GSEA [25,30] make no assumptions about variability or the form of the population distribution and are useful when the population distribution is not normal or unknown. However, because non-parametric tests use ranks instead of measured values, they tend to be less powerful, informative, and flexible than corresponding parametric tests [31].

As described earlier, the theoretical basis for using normal distribution in PAGE is Central Limit Theorem, which states that when sampling size n is large enough, distribution of an average of sampled observations is normal regardless of the nature of parent distribution. In statistics, sampling size of 30 is generally sufficient, although the actual sampling size fulfilling Central Limit Theorem is dictated by how parent distribution is close to normal distribution. In our case, sampled observations were fold changes in expression of randomly chosen genes in a microarray data set grouped into pre-defined randomly chosen gene sets. We found that sampling size of 10 was sufficient for demonstrating close to normal distribution of averages of fold changes of constituent genes in gene sets as inferred by several normality tests including Kolmogorov-Smirnov (Fig. 1C), Anderson-Darling and Cramer-von Mises (data not shown). In our opinion, the reason that normality analysis of microarray data sets can be performed with a much smaller sampling size (10) than generally required is because parent population of parameters, i.e., fold changes of all genes being compared in microarray data sets, is already somewhat close to normal. Indeed, most fold change values lie in the center position of the distribution and the proportion of significantly changed genes decreased along the axis to both directions (Fig. 1A).

It is clear that the statistical tools used in PAGE direct the program to analysis of pre-defined gene sets in microarray data sets rather than individual genes. This design was intentional. Regardless of the experimental paradigm, the majority of the cellular transcripts analyzed for differential expression on genome-wide microarray chips such as the Affymetrix 133A/B show statistically insignificant changes. For example, our analysis of gene expression profile of HIV-1-infected astrocytes on U133A/B detected about 740 different transcripts with fold changes of > 2 or < -2 and p ≤ 0.05 [32]. This result also means that the signals obtained with over 40,000 other probes on the chips in these experiments were not considered as significant. Thus, many potentially relevant but subtle changes in biological systems may not be readily detectable by individual gene analysis of differentially expressed gene lists. PAGE, like GSEA [25], attempts to resolve this problem by utilizing the phenomenon of gene co-regulation. In complex biological systems, many genes belonging to the same family and performing similar functions or genes acting in the same biological pathway are co-regulated. Conversely, in disease states, these genes may be coordinately dysregulated. Characterization of gene co-regulation (or co-dysregulation) under different physiological and pathological conditions is an important research problem that can now be approached by bioinformatics tools [33]. The assumption behind the gene set enrichment concept is that the statistical significance of coordinated changes in a set of co-regulated genes will be greater than that for individual genes in the set. This assumption was at least in part validated by applications of GSEA and seems to be borne out for PAGE as well (this work). In fact, we consider PAGE (as well as GSEA) not only as a program for detecting correlations between experimental conditions and changes in behavior of known gene sets containing co-regulated genes, but also as a tool for intentional search for novel gene co-regulation (or co-dysregulation) in microarray data sets as part of testable hypotheses.

It may be considered paradoxical to apply a statistical test based on normal distribution to an explicit goal of detecting sets of co-regulated, that is, interdependent genes. The normal distribution paradigm requires that sampled observations are independent and identically distributed, or IID. However, we would like to argue that gene dependency caused by co-regulation in a given microarray data set should be regarded as rare, and thus statistically significant. In developing this program, we started with a basic assumption, a null-hypothesis, that all genes in a given microarray data set are independent of each other and identically distributed, that is, they are not co-regulated. With given gene sets as testable hypotheses, we then tested whether there is a significant shift of behavior of genes as a group. When we observed a significant change in a given gene set, we rejected the null-hypothesis and concluded that those genes in a gene set are co-regulated and dependent on each other. We found that with the statistical tools we used, we matched and in most cases exceeded the ability of GSEA to detect co-regulated genes.

In a direct comparison of PAGE and GSEA using published data bases, the p-values of PAGE were lower than the respective p values obtained by GSEA and as a result, the number of gene sets that can be considered significantly changed was larger (Table 1 and see Additional files 1, 2, 3, 4). Similar results were obtained in an extensive simulation study (see Additional file 5). This confirms that similar to other applications [31] the parametric statistical test is more powerful than the non-parametric method when applied to gene set enrichment analysis Two other features of PAGE facilitate the computational process involved in running the program. First, because PAGE uses standard normal distribution as a background distribution, there is no need for the preceding permutation step required for this calculation in GSEA [25]. This reduces computation time at least 1,000 times when one performs 1,000 permutation of data set to get a background distribution. Secondly, the Z score of PAGE is two-tailed, showing gene sets of both increased and decreased expression in a single analysis. In one-tailed programs [11,12,25], the entire process from ranking gene lists to class permutation and statistical inference must be repeated after analysis in one direction. Thus PAGE is a statistically powerful gene set enrichment analysis tool with features that decrease the computational burden of such programs and increase the amount of information obtained per one analysis.

With wider availability of the gene microarray technology, there is an exponential increase in publicly accessible microarray data bases obtained on different platforms, by different laboratories, and addressing a variety of biological questions. A number of increasingly advanced data analysis tools have been developed to begin to compare and integrate this diverse and often incompatible information, including programs to identify biological themes instead of differentially expressed gene lists [12,25] or programs which identify significant genes displaying consistent changes across biologically different systems [15,24]. Each approach was shown to lead to better congruency among diverse data sets than would be achieved by direct comparison of data sets, whether in demonstrating common transcriptional profiles of prostate cancer [34], common molecular markers of lung cancer [15], or a common biological theme in diabetic muscle [25].

We have found that PAGE also can be applied to integrative data analysis across various microarray platforms and biological systems. As with GSEA [25] or EASE [12], the key to PAGE utility for this purpose is the ability of the program to compare microarray data sets for gene sets rather than individual genes. Our results indicate that PAGE works well with different probe level analysis methods (Table 2) and different microarray platforms (Table 3), in each case being able to identify several common biological pathways in the same starting material tested irrespective of the platform or primary analytical method used. Gene set analysis by PAGE was also far more discriminatory than individual gene analysis in finding common biological pathway changes in different microarray data sets generated to address the same biological question, the difference between young and aged muscles (Fig. 2 and Table 4). Another feature of PAGE that is useful for comparison of multiple microarray data sets is the Z score. Z score is a normalized and linear-scale value which is microarray platform independent and which is convenient to use as an input for subsequent analysis. It is possible to generate a data matrix containing Z scores of pre-defined gene sets of multiple data sets obtained on different microarray platforms and then perform cluster analysis to identify gene sets of specific interest or to identify relationships among data sets. We applied this approach recently to cluster analysis of multiple microarray data sets of macrophages infected with bacteria, protozoa, HIV-1, or treated with cytokines, and identified gene sets that were specifically changed in HIV-1-infected cells (S.-Y. Kim and M.J. Potash, unpublished), suggesting that the Z score system of PAGE will be useful for asking broad biological questions.

## Conclusion

The increasing use of microarrays comparing a carefully selected baseline to transformed tissue, differentiated tissue, or pathogen-infected tissue among others is creating a truly global database of gene expression profiles. Integrative analysis of this immense amount of information by programs such as EASE [12], meta-analysis of microarrays [15], or GSEA [25] begins to discern general patterns governing fundamental and disease-related biological processes. The program described here, parametric analysis of gene set enrichment analysis or PAGE is statistically sensitive and requires less computation than many other programs. PAGE identified significantly changed biological themes from microarray data set irrespective of microarray data analysis methods or microarray platforms. PAGE identified more common biological themes in different microarray data sets addressing the same biological problem than analysis of individual gene level. Finally, the Z score of PAGE is a normalized, linear scale value that can be used in subsequent meta-analysis. We offer PAGE as a useful microarray analysis tool.

## Methods

### Data sets

The microarray data set, GSEA results for NGT versus DM2, and probe sets corresponding to gene sets of Mootha *et al. *[25] were downloaded from the authors' website [35]. Microarray data sets of the muscle function and aging studies (GDS 287 and GDS 472) [36,37], Duchenne muscular dystrophy (DMD) study (GDS 563) and breast cancer cell line experiment (GSE 1299) [20] were downloaded from Gene Expression Omnibus [38] website.

### Analysis of Microarray Data

Each Affymetrix microarray data set was normalized to have mean expression value of 1,000. Expression values below 100 were floored to 100 and then all expression values were transformed by logarithm base two. Fold change between two experimental groups was measured to identify differentially expressed genes and unpaired t-test was used to infer statistical significance of differentially expressed genes.

### Parametric Analysis of Gene Set Enrichment (PAGE)

We created predefined gene sets for major mammalian Affymetrix platforms (human: U133A, U95A, HuFL6800; mouse: M74Av2; and rat: U34) from corresponding Affymetrix annotation files [39] with gene ontology (GO) biological processes, GO cellular components, GO molecular functions, and pathways as the main categories.

Z score for each gene set was calculated as follows. First, from input data containing fold change values for each genes between two experimental groups, mean of total fold change values (μ) and standard deviation of total fold change values (δ) of a given microarray data set were calculated. Then, when the mean of fold change values of genes for a given gene set was Sm and the size of a given gene set was m, the Z score was calculated as Z = (Sm – μ)*m^1/2 ^/ δ

The statistical tools within the Microsoft Excel program were used to calculate p-values from Z scores. We used an R package nortest [40] to test whether a given distribution is different from standard normal distribution or not. The R statistical programming language [40] was used for general statistical analysis and computing.

### Implementation of PAGE

PAGE was written in the freely available Python programming language [41] applicable to most computer platforms and operating systems including Windows, Macintosh, and LINUX/UNIX. We also prepared stand-alone Windows-executable PAGE program using py2exe tool [42].

## Availability and requirements

• Project name: PAGE

• Project home page: In construction

• Operating system: Platform independent

• Programming language: Python, Windows-executable through py2exe [42]

• Other requirements: None

• License: None

• Any restrictions to use by non-academics: None

• The Python script PAGE is available from SYK upon request

• The beta version of GSEA is now available at [43]

## Authors' contributions

SYK designed and implemented PAGE method, performed the bioinformatics analyses, and drafted the manuscript. DJV supervised the development of PAGE method and wrote the manuscript. All authors read and approved the final manuscript.

## Supplementary Material

Additional File 1 Comparison of GDS 472 by PAGE and GSEA: Ranking by GSEA. Click here for file

Additional File 2 Comparison of GDS 287 by PAGE and GSEA: Ranking by GSEA. Click here for file

Additional File 3 Comparison of GDS 963n1 by PAGE and GSEA: Ranking by GSEA. Click here for file

Additional File 4 Comparison of GDS 963n1 by PAGE and GSEA: Ranking by PAGE. Click here for file

Additional File 5 Simulation study comparing statistical sensitivity PAGE and GSEA. Click here for file
