# Supplementary material for: PAGE: Parametric Analysis of Gene Set Enrichment
Source: BMC Bioinformatics. 2005 Jun 8;6:144. doi: 10.1186/1471-2105-6-144 (PMC1183189; doi:10.1186/1471-2105-6-144)
Supplement: Additional File 1 — Comparison of GDS 472 by PAGE and GSEA: Ranking by GSEA. [file 1471-2105-6-144-S1.pdf]

Table A1. Comparison of GDS 472 by PAGE and GSEA: Ranking by GSEA

Gene Set	PAGE Z score	p-value	GSEA ES	NES	p-value
muscle_myosin	-4.0327	5.51E-05	-0.6703	-1.5934	0.0000
cell_cycle_arrest	-2.4142	0.0158	-0.4117	-1.3109	0.0625
radiation_sensitivity	-1.8167	0.0693	-0.4217	-1.2465	0.0755
P53_UP	-4.0146	5.95E-05	-0.5139	-1.3192	0.0816
p53_signalling	-2.8896	0.0039	-0.3306	-1.2340	0.0816
etsPathway	-2.8058	0.0050	-0.5217	-1.3080	0.1053
raccycdPathway	-1.3869	0.1655	-0.3583	-1.2005	0.1132
carm-erPathway	-2.1483	0.0317	-0.3821	-1.2382	0.1333
atmPathway	-2.4814	0.0131	-0.4639	-1.2033	0.1471
P53_DOWN	-1.0935	0.2742	-0.3831	-1.1541	0.1667
CR_IMMUNE_FUNCTION	-0.3974	0.6911	-0.3268	-1.1452	0.1837
mRNA_splicing	-2.2718	0.0231	-0.3329	-1.1813	0.2000
HOXA9_UP	-2.3688	0.0178	-0.3876	-1.2367	0.2154
CR_TRANSCRIPTION_FACTORS	-1.7588	0.0786	-0.3249	-1.1084	0.2162
tumor_supressor	-1.9976	0.0458	-0.3624	-1.1211	0.2292
MAP00531_Glycosaminoglycan_degradation	-1.3951	0.1630	-0.4409	-1.2251	0.2321
DNA_DAMAGE_SIGNALLING	-2.8829	0.0039	-0.3396	-1.1629	0.2364
tnfr1Pathway	-2.3683	0.0179	-0.4316	-1.2133	0.2407
g2Pathway	-1.7803	0.0750	-0.4261	-1.1835	0.2500
nkcellsPathway	-0.9083	0.3637	-0.4169	-1.0992	0.2667
tnf_and_fas_network	-1.0109	0.3121	-0.3823	-1.0924	0.2754
caspasePathway	-2.1302	0.0332	-0.4702	-1.1471	0.2979
gsk3Pathway	-0.9618	0.3362	-0.3298	-1.0634	0.3051
p53Pathway	-1.5284	0.1264	-0.3861	-1.0904	0.3103
mRNA_processing	-3.5606	0.0004	-0.3729	-1.1319	0.3134
MAP00500_Starch_and_sucrose_metabolism	0.5361	0.5919	-0.3408	-1.1099	0.3333
mitochondriaPathway	-0.7847	0.4327	-0.4164	-1.0881	0.3469
keratinocytePathway	-1.2659	0.2056	-0.3327	-1.0540	0.3607
d4gdiPathway	-2.2048	0.0275	-0.4968	-1.1358	0.3617
p53hypoxiaPathway	-0.8010	0.4231	-0.3406	-1.0347	0.3654
