# Supplementary material for: PAGE: Parametric Analysis of Gene Set Enrichment
Source: BMC Bioinformatics. 2005 Jun 8;6:144. doi: 10.1186/1471-2105-6-144 (PMC1183189; doi:10.1186/1471-2105-6-144)
Supplement: Additional File 2 — Comparison of GDS 287 by PAGE and GSEA: Ranking by GSEA. [file 1471-2105-6-144-S2.pdf]

Table A2. Comparison of GDS 287 by PAGE and GSEA: Ranking by GSEA

Gene Set	PAGE Z score	p-value	GSEA ES	NES	p-value
p53hypoxiaPathway	-4.5002	6.79E-06	-0.4718	-1.6393	0.0000
DNA_DAMAGE_SIGNALLING	-3.0801	0.0021	-0.2717	-1.3045	0.0000
cell2cellPathway	-2.7031	0.0069	-0.5589	-1.6968	0.0156
HOXA9_DOWN	-4.3054	1.67E-05	-0.4235	-1.6004	0.0161
nkcellsPathway	-3.0887	0.0020	-0.4700	-1.5672	0.0179
p53Pathway	-3.0569	0.0022	-0.4149	-1.4027	0.0238
cell_cycle_arrest	-2.7869	0.0053	-0.3798	-1.4769	0.0267
mRNA_processing	-4.9809	6.33E-07	-0.4546	-1.5564	0.0303
INSULIN_2F_UP	-6.4688	9.88E-11	-0.3410	-1.4772	0.0308
atmPathway	-4.1716	3.03E-05	-0.4543	-1.5335	0.0308
cell_cycle_checkpoint	-2.3224	0.0202	-0.3807	-1.5321	0.0328
mRNA_splicing	-3.9571	7.59E-05	-0.4001	-1.4710	0.0400
LEU_UP	-3.5795	0.0003	-0.2493	-1.2396	0.0577
eponfkbPathway	-3.6026	0.0003	-0.4227	-1.3801	0.0600
p53_signalling	-2.8926	0.0038	-0.2651	-1.2563	0.0682
HOXA9_UP	-3.3693	0.0008	-0.3992	-1.4511	0.0685
XINACT	-1.6032	0.1089	-0.3595	-1.3885	0.0784
Proteasome_Degradation	-3.5819	0.0003	-0.4008	-1.5300	0.0806
CR_CYTOSKELETON	-3.3495	0.0008	-0.4317	-1.3630	0.0822
tidPathway	-2.3461	0.0190	-0.3696	-1.3025	0.0862
muscle_myosin	-5.1167	3.11E-07	-0.4315	-1.3197	0.0984
ucalpainPathway	-1.6543	0.0981	-0.3498	-1.2444	0.1020
g2Pathway	-2.6938	0.0071	-0.3765	-1.2905	0.1061
carm-erPathway	-1.4253	0.1541	-0.3143	-1.2644	0.1081
hivnefPathway	-3.4484	0.0006	-0.2954	-1.2734	0.1094
shhPathway	-1.5605	0.1187	-0.3976	-1.2872	0.1207
proteasomePathway	-2.0807	0.0375	-0.4161	-1.4494	0.1250
pdgfPathway	-3.3477	0.0008	-0.3999	-1.3494	0.1333
telPathway	-2.9998	0.0027	-0.3769	-1.3092	0.1333
ccr5Pathway	-3.2935	0.0010	-0.3228	-1.2279	0.1455
