# Supplementary material for: PAGE: Parametric Analysis of Gene Set Enrichment
Source: BMC Bioinformatics. 2005 Jun 8;6:144. doi: 10.1186/1471-2105-6-144 (PMC1183189; doi:10.1186/1471-2105-6-144)
Supplement: Additional File 3 — Comparison of GDS 963n1 by PAGE and GSEA: Ranking by GSEA. [file 1471-2105-6-144-S3.pdf]

Table A3. Comparison of GDS 963n1 by PAGE and GSEA: Ranking by GSEA

Gene Set	PAGE Z score	p-value	ES	GSEA NES	p-value
MAP00280_Valine_leucine_and_isoleucine_degradation	3.6010	0.0003	0.5468	1.7513	0.0222
MAP00120_Bile_acid_biosynthesis	2.3297	0.0198	0.5674	1.7058	<0.01
GLUCOSE_UP	4.0669	0.0000	0.4416	1.6302	<0.01
LEU_UP	3.6744	0.0002	0.3609	1.6154	<0.01
Inflammatory_Response_Pathway	3.0751	0.0021	0.5419	1.6142	0.0508
cell_adhesion	6.5012	0.0000	0.3979	1.5749	<0.01
MAP00260_Glycine_serine_and_threonine_metabolism	2.7381	0.0062	0.4660	1.4970	0.0192
MAP00252_Alanine_and_aspartate_metabolism	2.7539	0.0059	0.4577	1.4779	0.1053
CR_CYTOSKELETON	2.9006	0.0037	0.4408	1.4613	0.0345
pgc1aPathway	0.8129	0.4163	0.3701	1.4527	<0.01
calcineurinPathway	0.9167	0.3593	0.4042	1.4473	0.0227
ST_Wnt_Ca2_cyclic_GMP_Pathway	2.2348	0.0254	0.4886	1.4350	0.0588
CR_CAM	2.8259	0.0047	0.3468	1.4316	0.0192
FA	1.7671	0.0772	0.4533	1.4210	0.1667
intrinsicPathway	1.7768	0.0756	0.4435	1.3683	0.1607
integrinPathway	0.9518	0.3412	0.3744	1.3642	0.1600
ccr5Pathway	1.3963	0.1626	0.3566	1.3542	0.1321
MAP00350_Tyrosine_metabolism	2.1392	0.0324	0.4092	1.3385	0.1207
ndkDynaminPathway	0.7258	0.4679	0.3832	1.3280	0.1522
ANTI_CD44_UP	1.1332	0.2572	0.3812	1.3158	0.0652
MAP00640_Propanoate_metabolism	1.5603	0.1187	0.4051	1.3122	0.1316
il7Pathway	2.2041	0.0275	0.3862	1.3116	0.1667
ecmPathway	1.3016	0.1931	0.3588	1.3102	0.0882
mcalpainPathway	1.1795	0.2382	0.3936	1.2782	0.1887
GLUT_UP	4.1735	0.0000	0.2481	1.2705	0.1250
EMT_UP	2.9125	0.0036	0.3859	1.2493	0.2439
rhoPathway	1.7229	0.0849	0.3292	1.2394	0.1961
cell_adhesion_receptor_activity	2.9385	0.0033	0.3510	1.2283	0.1731
FRASOR_ER_DOWN	0.5235	0.6007	0.2940	1.1861	0.2500
EMT_DOWN	1.8760	0.0607	0.3194	1.1780	0.2128
mef2dPathway	0.1754	0.8607	0.3153	1.1521	0.2549
HTERT_DOWN	0.9126	0.3615	0.2601	1.1169	0.2353
