# Supplementary material for: PAGE: Parametric Analysis of Gene Set Enrichment
Source: BMC Bioinformatics. 2005 Jun 8;6:144. doi: 10.1186/1471-2105-6-144 (PMC1183189; doi:10.1186/1471-2105-6-144)
Supplement: Additional File 4 — Comparison of GDS 963n1 by PAGE and GSEA: Ranking by PAGE. [file 1471-2105-6-144-S4.pdf]

Table A4. Comparison of GDS 963n1 by PAGE and GSEA: Ranking by PAGE

Gene Set	PAGE		GSEA		
	Z score	p-value	ES	NES	p-value
cell_adhesion	6.5012	0.0000	0.3979	1.5749	<0.01
GLUT_UP	4.1735	0.0000	0.2481	1.2705	0.1250
GLUCOSE_UP	4.0669	0.0000	0.4416	1.6302	<0.01
LEU_UP	3.6744	0.0002	0.3609	1.6154	<0.01
MAP00280_Valine_leucine_and_isoleucine_degradation	3.6010	0.0003	0.5468	1.7513	0.0222
cell_adhesion_molecule_activity	3.5182	0.0004	0.2933	1.1291	0.3214
Inflammatory_Response_Pathway	3.0751	0.0021	0.5419	1.6142	0.0508
cell_adhesion_receptor_activity	2.9385	0.0033	0.3510	1.2283	0.1731
EMT_UP	2.9125	0.0036	0.3859	1.2493	0.2439
CR_CYTOSKELETON	2.9006	0.0037	0.4408	1.4613	0.0345
CR_CAM	2.8259	0.0047	0.3468	1.4316	0.0192
MAP00252_Alanine_and_aspartate_metabolism	2.7539	0.0059	0.4577	1.4779	0.1053
MAP00260_Glycine_serine_and_threonine_metabolism	2.7381	0.0062	0.4660	1.4970	0.0192
MAP00120_Bile_acid_biosynthesis	2.3297	0.0198	0.5674	1.7058	<0.01
ST_Wnt_Ca2_cyclic_GMP_Pathway	2.2348	0.0254	0.4886	1.4350	0.0588
il7Pathway	2.2041	0.0275	0.3862	1.3116	0.1667
MAP00350_Tyrosine_metabolism	2.1392	0.0324	0.4092	1.3385	0.1207
