# Supplementary material for: PAGE: Parametric Analysis of Gene Set Enrichment
Source: BMC Bioinformatics. 2005 Jun 8;6:144. doi: 10.1186/1471-2105-6-144 (PMC1183189; doi:10.1186/1471-2105-6-144)
Supplement: Additional File 5 — Simulation study comparing statistical sensitivity PAGE and GSEA. [file 1471-2105-6-144-S5.pdf]

Table A5: Simulation study comparing statistical sensitivity PAGE and GSEA

| | PAGE | | GSEA | |
|---|---|---|---|---|
| Mean Difference | Z score | p-value | ES | p-value |
| 1 | 8.227 | 2.22E-16 | 128.54 | 0.001 |
| 0.95 | 8.055 | 8.88E-16 | 152.8663 | 0.001 |
| 0.9 | 8.522 | 0 | 112.3632 | 0.001 |
| 0.85 | 7.338 | 2.18E-13 | 133.268 | 0.003 |
| 0.8 | 6.706 | 2.01E-11 | 138.0922 | 0.001 |
| 0.75 | 5.232 | 1.68E-07 | 127.3383 | 0.001 |
| 0.7 | 6.932 | 4.18E-12 | 91.96096 | 0.001 |
| 0.65 | 7.067 | 1.59E-12 | 86.73476 | 0.001 |
| 0.6 | 5.078 | 3.82E-07 | 138.2932 | 0.001 |
| 0.55 | 6.071 | 1.28E-09 | 41.20655 | 0.014 |
| 0.5 | 4.481 | 7.44E-06 | 79.599 | 0.002 |
| 0.45 | 5.203 | 1.96E-07 | 20.00025 | 0.136 |
| 0.4 | 3.957 | 7.59E-05 | 60.70428 | 0.003 |
| 0.35 | 2.603 | 0.009241 | 23.71889 | 0.0555 |
| 0.3 | 2.132 | 0.033007 | 3.316625 | 0.7625 |
| 0.25 | 3.216 | 0.0013 | 7.939799 | 0.415 |
| 0.2 | 1.408 | 0.159131 | 25.9288 | 0.0675 |
| 0.15 | 1.711 | 0.087081 | 0 | 0.994 |
| 0.1 | 0.753 | 0.45145 | 9.04534 | 0.34 |
| 0.05 | -0.289 | 0.772582 | 9.54786 | 0.3785 |

Simulation procedure for comparing statistical sensitivity of PAGE and GSEA. We created two sample groups of data sets, the experimental group (exp; size n=10) and the control group (cont; size n=10). Each data set within the group consisted of expression values of 2,000 genes. 1,980 of these were chosen randomly from standard normal distribution presuming no difference between cont and exp groups. The remaining, randomly chosen 20 values were assigned as a gene set of interest. The mean expression values of genes in the exp gene sets were then increased relative to cont by values from 0.05 to 1.0 in increments of 0.05, to simulate small differences in mean expression values between exp and cont data sets. The data sets were analyzed by PAGE and GSEA for Z scores and p-values and ES and p-values, respectively. As shown in the Table, PAGE detected test gene set as significant when mean expression difference between exp and cont was 0.25 or above; the respective limit of detection for GSEA was 0.4 to 0.5. The p-values of PAGE were lower than the respective p-values of GSEA over the entire range of mean differences tested.
